# A Screening Method Using Anomaly Detection on a Smartphone for Patients With Carpal Tunnel Syndrome: Diagnostic Case-Control Study

**DOI:** 10.2196/26320

**Published:** 2021-03-14

**Authors:** Takafumi Koyama, Shusuke Sato, Madoka Toriumi, Takuro Watanabe, Akimoto Nimura, Atsushi Okawa, Yuta Sugiura, Koji Fujita

**Affiliations:** 1 Department of Orthopedic and Spinal Surgery Graduate School of Medical and Dental Sciences Tokyo Medical and Dental University Tokyo Japan; 2 School of Science for Open and Environmental Systems Graduate School of Science and Technology Keio University Kanagawa Japan; 3 Department of Functional Joint Anatomy Graduate School of Medical and Dental Sciences Tokyo Medical and Dental University Tokyo Japan

**Keywords:** carpal tunnel syndrome, anomaly detection, machine learning, smartphone, screening, thumb, diagnostic, data collection, app, algorithm

## Abstract

**Background:**

Carpal tunnel syndrome (CTS) is a medical condition caused by compression of the median nerve in the carpal tunnel due to aging or overuse of the hand. The symptoms include numbness of the fingers and atrophy of the thenar muscle. Thenar atrophy recovers slowly postoperatively; therefore, early diagnosis and surgery are important. While physical examinations and nerve conduction studies are used to diagnose CTS, problems with the diagnostic ability and equipment, respectively, exist. Despite research on a CTS-screening app that uses a tablet and machine learning, problems with the usage rate of tablets and data collection for machine learning remain.

**Objective:**

To make data collection for machine learning easier and more available, we developed a screening app for CTS using a smartphone and an anomaly detection algorithm, aiming to examine our system as a useful screening tool for CTS.

**Methods:**

In total, 36 participants were recruited, comprising 36 hands with CTS and 27 hands without CTS. Participants controlled the character in our app using their thumbs. We recorded the position of the thumbs and time; generated screening models that classified CTS and non-CTS using anomaly detection and an autoencoder; and calculated the sensitivity, specificity, and area under the curve (AUC).

**Results:**

Participants with and without CTS were classified with 94% sensitivity, 67% specificity, and an AUC of 0.86. When dividing the data by direction, the model with data in the same direction as the thumb opposition had the highest AUC of 0.99, 92% sensitivity, and 100% specificity.

**Conclusions:**

Our app could reveal the difficulty of thumb opposition for patients with CTS and screen for CTS with high sensitivity and specificity. The app is highly accessible because of the use of smartphones and can be easily enhanced by anomaly detection.

## Introduction

Carpal tunnel syndrome (CTS) is a medical condition caused by compression of the median nerve in the carpal tunnel due to aging or hand overuse [1]. Patients with CTS develop numbness from the thumb to the ring finger and, in severe cases, thenar muscle atrophy [2]. Because the symptoms impair thumb motions, CTS can impede everyday movements, such as holding a pen or chopsticks and handling buttons on clothes [3,4]. The prevalence of CTS is approximately 2% to 14%, and it affects more women than men [5,6]. Since most patients with CTS are aged 40 years or older [5,6] and the number of older people is increasing worldwide, the number of patients with CTS is expected to increase. Nonsurgical therapy, such as a wrist brace [7] or steroid injection into the carpal tunnel [8], is typically prescribed, but surgery is often necessary for severe symptoms [2]. Patients often delay seeking medical attention until the numbness worsens and thenar atrophy develops. The symptoms in severe cases recover slowly postoperatively [9,10]; therefore, early diagnosis of CTS and surgery before the symptoms worsen is important.

Physical findings, such as the Tinel sign or Phalen test, may be used; however, their sensitivity and specificity are not high [11,12]. Although a nerve conduction study (NCS) is considered useful for diagnosing CTS [13,14], the equipment is expensive and the process can be painful and long (up to an hour). In addition, a skilled technician must perform the detailed NCS [15]. Due to impaired access to NCSs, diagnosis is largely performed subjectively by doctors in clinics and small hospitals in which there are neither hand surgeons nor specialized equipment, contributing to delayed diagnosis.

In recent years, cameras and sensors in mobile devices have become smaller and more sophisticated and can now measure the state of the user. Various studies have been conducted on the use of mobile devices to acquire physical information and diagnose diseases [16-18]. Fujita et al [19] developed an app for screening CTS with an accuracy of 83% using a tablet and support vector machine, utilizing a machine learning technique. However, as patients with CTS are mainly aged 40 years and older and the rate of use of tablets in this age group is not high [20,21], the system may be difficult to introduce. Furthermore, the machine learning for the binary classification used in the aforementioned app needs 2 data sets, both from healthy controls and from patients. Large data sets are needed to enhance machine learning; however, these are difficult to collect due to the low prevalence of CTS.

To address these concerns, we developed a screening app for CTS using a smartphone and an anomaly detection algorithm [22] because the usage rate of smartphones is higher than that of tablets [20,21] and anomaly detection algorithms need only easily collected data sets of healthy controls. We aimed to examine whether our system was a useful screening tool for CTS.

## Methods

### Recruitment

This study was approved by the Institutional Review Board of Tokyo Medical and Dental University. Written informed consent was provided by all participants.

We recruited 21 preoperative patients (36 hands) with CTS at the Tokyo Medical and Dental University Hospital as the CTS group and 15 healthy volunteers (27 hands) at an osteopathic clinic as the non-CTS group from July 2018 to May 2019. Experienced hand surgeons diagnosed CTS based on symptoms, physical findings such as the Tinel sign and Phalen test, x-ray images of the hands, and NCSs measured by Neuropack X1 (Nihon Kohden). Patients were classified based on the Bland classification [23]. Patients with a history of other hand injury or surgery, recurrence after release surgery of the carpal tunnel, positive imaging findings indicative of first carpometacarpal or thumb metacarpophalangeal osteoarthritis (which could affect thumb motion), or suspicion of a disease of the cervical spine were excluded. In the non-CTS group, volunteers were excluded if they had a history of wrist, hand, or finger disease, injury, or surgery; finger numbness; thumb pain; or positive physical findings of CTS.

### App Design

**Figure 1:**
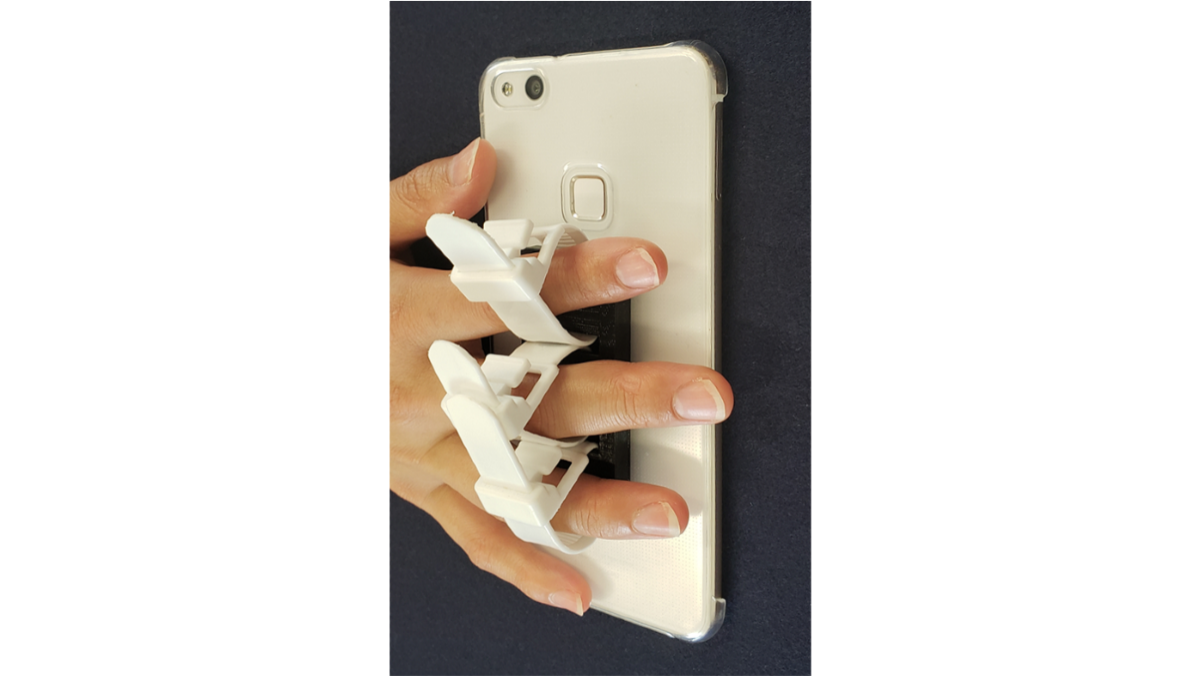
A finger guide attached to the back of the smartphone to fix the position of the fingers other than the thumb.

We used a Huawei P10 Lite (Huawei Technologies) phone and developed the app using Unity software (Unity Technologies). We also created a finger guide, which was attached to the back of the smartphone to fix the position of the fingers other than the thumb (Figure 1). The guide consisted of a component created with a 3D printer and 3 binding bands. The length of the binding band could be adjusted to adapt to the participant’s finger thickness.

**Figure 2:**
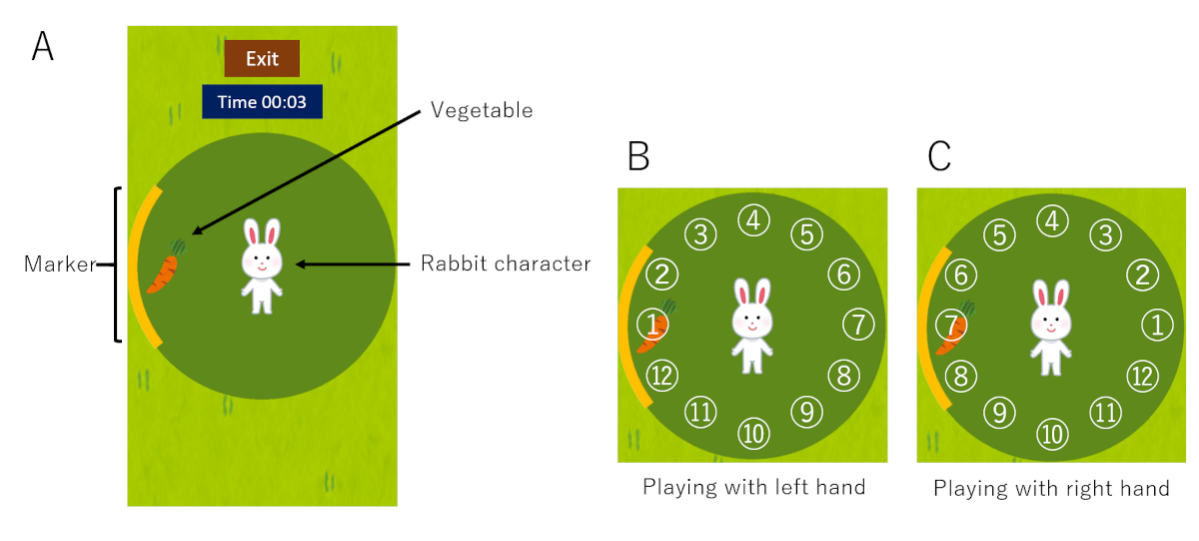
The images of the app. A rabbit character and vegetables were displayed in the green circle. Vegetables were located at the center or edge of the circle, and markers were also displayed when the vegetables were located at the edge (A). Vegetables appeared in 12 numbered directions, and the numbers were reversed depending on whether the player used the left (B) or right (C) hand.

**Figure 3:**
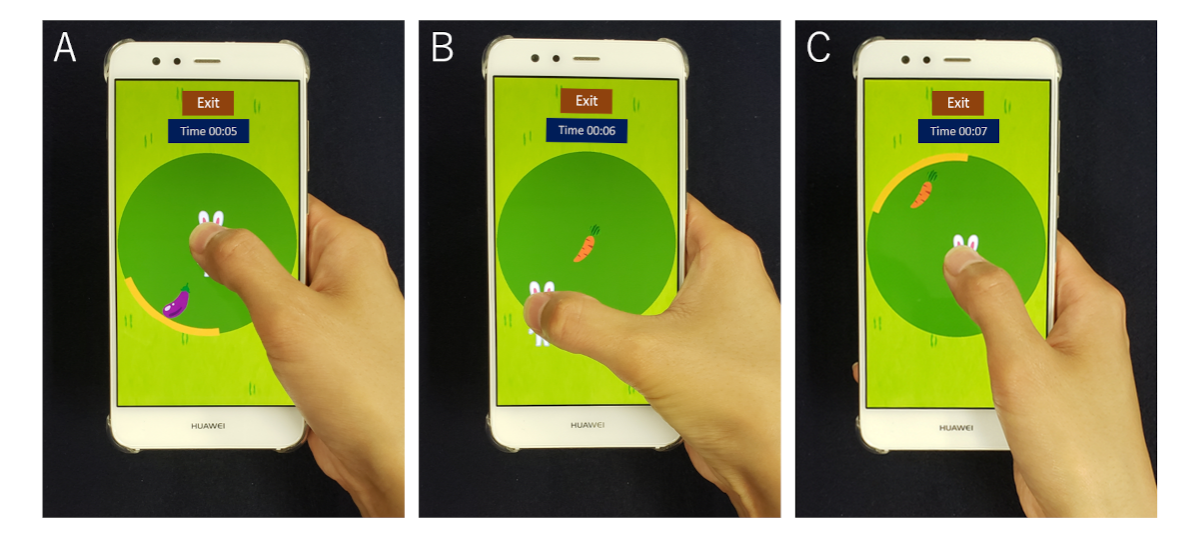
The images of the app while playing the game. The player touched and controlled a rabbit character with the thumb of each hand to collect vegetables. Vegetables appeared in one of 12 directions (A). When each vegetable was collected, the next appeared alternately at the center of the circle (B) or in another direction (C).

In this app, the player controlled a rabbit character with their thumb and collected vegetables (carrots, radishes, or eggplants) that appeared on the screen (screen A in Figure 2). When vegetables were hidden by the thumb, broad markers that indicated the direction of the vegetable were also displayed. The vegetables appeared sequentially in 12 directions along a circle with a 2-cm radius in a random order (screens B and C in Figure 2). The vegetables appeared alternatively in one of 12 directions and at the center, and the user collected them in each direction and in the center in turn (Figure 3). If a vegetable was not collected within 5 seconds, it disappeared and then reappeared at another place. In the practice phase, vegetables appeared randomly in 4 directions. Subsequently, in the measurement phase, the game ended after 2 sequences of vegetable appearances in 12 directions. The participants played the game in the app twice. The position of the thumb and the time were recorded, and the average time, average velocity, and maximum velocity of the thumb movement for the 12 directions were calculated.

### Statistical Analysis

We used 2-tailed Student *t* tests to compare the age of participants, average time, average velocity, and maximum velocity of the thumb movement for the 12 directions between the non-CTS and CTS groups. Chi-square tests were used to compare sex, playing side of the hand, and hand dominance between the non-CTS and CTS groups. A *P* value below .05 was considered to indicate statistical significance.

**Figure 4:**
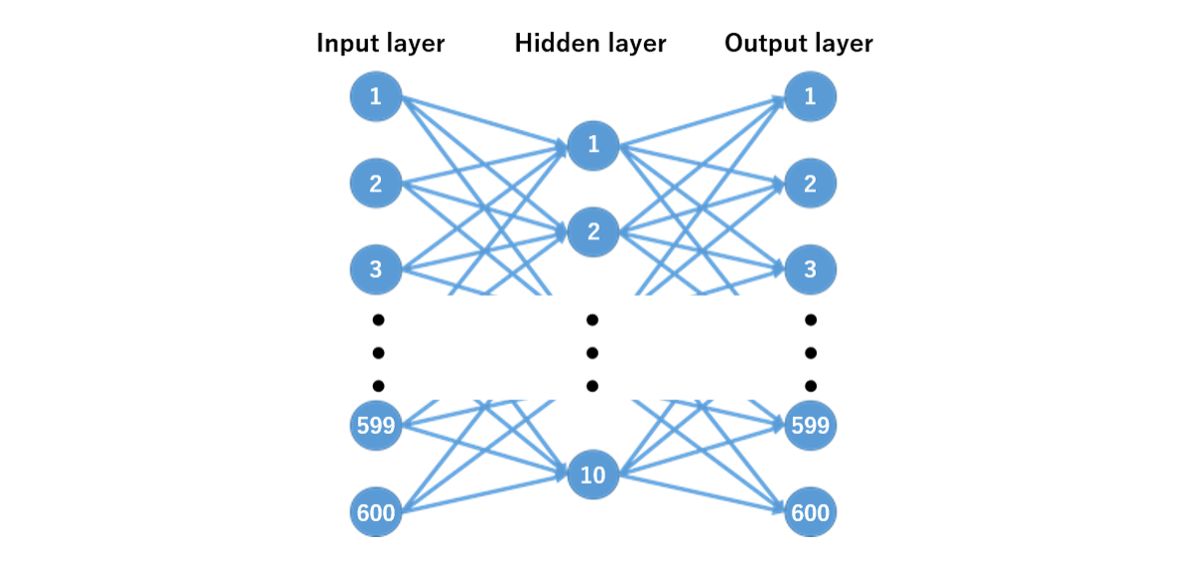
An image demonstrating how the autoencoder works. In our model, the input layer was 600 dimensions, the intermediate layer was 10 dimensions, and the output layer was 600 dimensions.

To generate a screening model that classified participants as CTS and non-CTS, we analyzed data sets using anomaly detection and an autoencoder (AE) [24,25]. Anomaly detection is the process of identifying data that differ from the norm in a data set. It has the advantage that it can be learned from normal group data only. The AE is a type of neural network with a 3-layer structure consisting of input, hidden, and output layers (Figure 4). The transformation of the input layer to the hidden layer is the encoder, and the attempted reconstruction of the hidden layer to the output layer constitutes the decoder. The AE performs unsupervised learning and is trained to reconstruct the input patterns. By reducing the number of units in a hidden layer compared with the number of units in the input layer, it enables dimensional compression.

**Figure 5:**
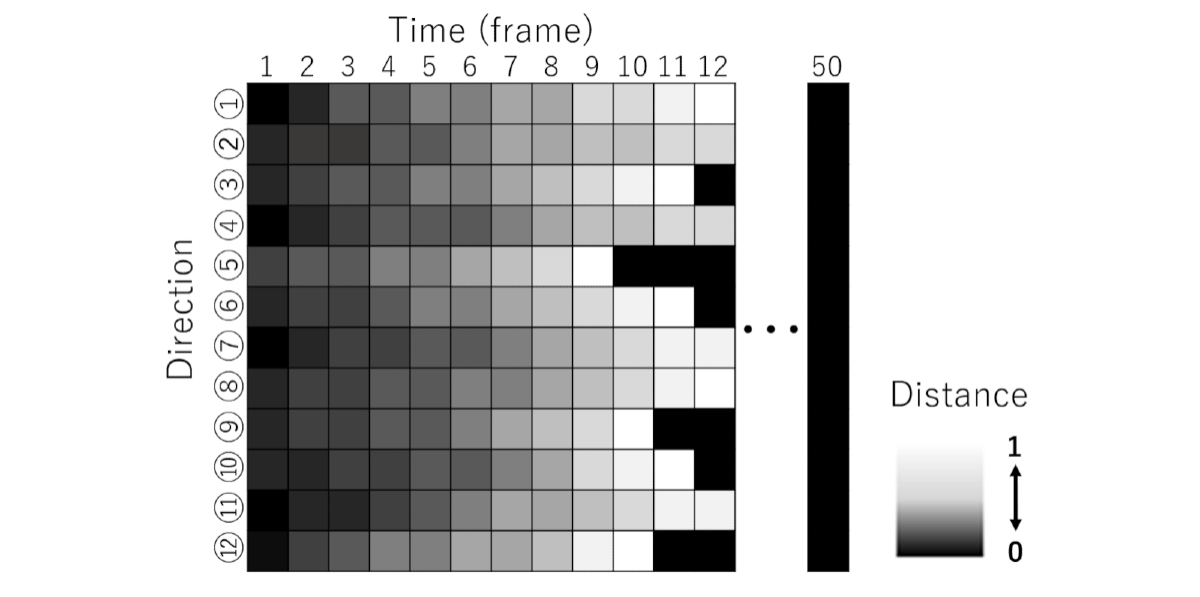
Grayscale image generated by the pixel values with the vertical axis set as each direction and the horizontal axis set as time. The intensity of the pixel was defined by the distance between the thumb and the center; the greater the distance, the lighter the intensity. Pixels of the frames when the thumb reached the circumference (vegetables) were white, and all pixels to the right of the frames were set to be filled with black. The vertical axis was set as 12 directions and the horizontal axis was set at a fixed time (50 frames).

First, we calculated the distance to the center of the screen from the coordinate data and converted this into a value from 0 to 1. In our proposed model, the first lap was only played as practice for the participants to get used to the app, and only the second-lap data were used for the analysis. Next, a grayscale image was generated by arranging the pixel values with the vertical axis set as each direction and the horizontal axis set as time (Figure 5). The horizontal axis consisted of 5 seconds, which is equal to 50 frames because the sampling rate was 10 Hz. Hence, the pixel count of the grayscale image was 600 pixels (12 directions × 50 frames). Finally, we validated the classification of non-CTS and CTS using the AE. For the AE, the 600 pixels of the grayscale image were used as the input layer, the intermediate layer was fixed at 10 dimensions, and the output layer was set to 600 dimensions (Figure 4).

We used the data from 12 hands in the non-CTS group for the training of the AE and validated them with the data from the 36 hands in the CTS group and 15 hands in the non-CTS group that were not used for the training. The reconstruction error of the AE was calculated using the mean square error of the difference between the input and output. By training the AE on non-CTS data only, we could detect patients with CTS because the reconstruction error was smaller for non-CTS data and larger for CTS data. We generated a receiver operating characteristic (ROC) curve by adjusting the cutoff value of the mean square error and calculated the area under the curve (AUC). The optimal cutoff value was set at the point where the Youden index was at its maximum in the ROC curve. Furthermore, to investigate which directional movements contribute to the diagnosis of CTS, we also generated modified screening models that classified CTS and non-CTS using data from only 4 consecutive directions of the 12 directions and calculated the AUC in the same way as above.

## Results

The characteristics of the participants are summarized in Table 1. There was no significant difference between the groups in terms of age, sex, or side of the playing hand.

**Table 1:** Characteristics of participants in the CTS and non-CTS groups.

Characteristic		Non-CTS^a^	CTS	*P* value
Participants, n		15	21	N/A^b^
Age (years), mean (SD)		63.5 (17.6)	64.3 (12.2)	.87
Sex (female), n		12	16	.63
Hand dominance (right), n		15	21	>.99
Hands, n		27	36	N/A
Side (right), n		15	17	.69
**Bland classification, n**				N/A
	Grade 1	N/A	5	
	Grade 2	N/A	6	
	Grade 3	N/A	15	
	Grade 4	N/A	0	
	Grade 5	N/A	9	
	Grade 6	N/A	1	

^a^CTS: carpal tunnel syndrome.

^b^N/A: not applicable.

**Figure 6:**
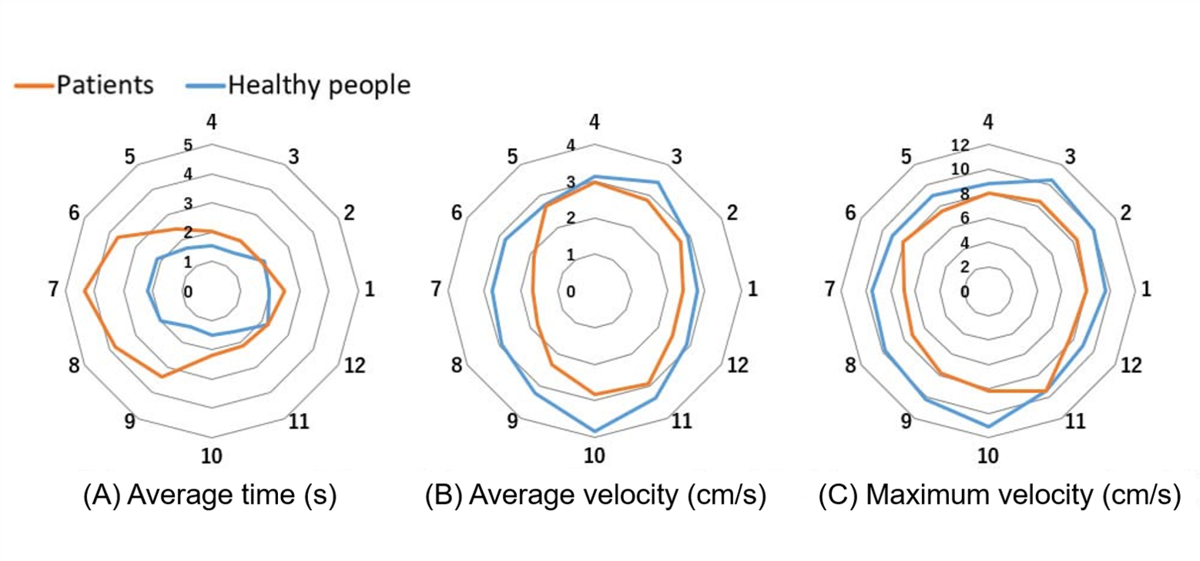
Representation of the average time taken to collect vegetables (A) and the average (B) and maximum (C) velocities in each direction.

Figure 6 shows the average time taken to collect vegetables and the average and maximum velocities in each direction. Compared with healthy people, the patients took significantly longer to collect the vegetable in directions 6 to 9, and both the average and maximum velocities of the patients were significantly slower in all directions.

**Figure 7:**
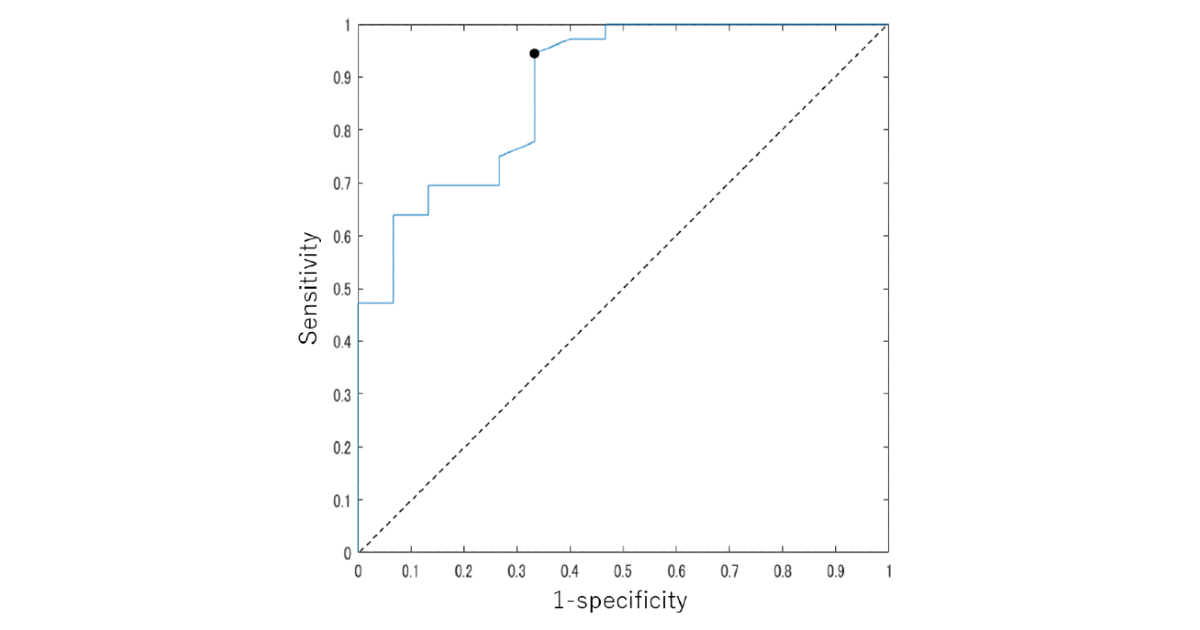
ROC curve of the screening model. The area under the ROC curve was 0.86. The black point indicates the optimal cutoff value, and the sensitivity and specificity at that point were 0.94 and 0.67, respectively. ROC: receiver operating characteristic.

The results of the screening model are shown in Table 2. The participants with and without CTS were classified with 94% sensitivity and 67% specificity. The ROC curve of the classification model is shown in Figure 7; the AUC was 0.86.

**Table 2:** The result of the screening model. People with and without CTS were classified with 94% sensitivity and 67% specificity.

True label	Predicted label, n	
	Non-CTS^a^	CTS
Non-CTS	10	5
CTS	2	34

^a^CTS: carpal tunnel syndrome.

The results of the modified screening models are shown in Table 3. The model using data from directions 8 to 11 had the highest AUC of 0.99 and could classify patients as CTS and non-CTS with 92% sensitivity and 100% specificity.

**Table 3:** The index of the modified screening models.

Direction^a^	Sensitivity, %	Specificity, %	AUC^b^
1-4	78	73	0.85
2-5	89	93	0.96
3-6	83	80	0.87
4-7	100	87	0.92
5-8	94	73	0.86
6-9	89	80	0.86
7-10	86	87	0.92
8-11	92	100	0.99
9-12	92	100	0.98
10-1	92	87	0.94
11-2	92	80	0.86
12-3	73	73	0.79

^a^Directions are based on screens B and C of Figure 2.

^b^AUC: area under the curve.

## Discussion

### Principal Results

In this study, we developed a smartphone app with a high ability to screen for CTS. The app could diagnose CTS with 94% sensitivity and 67% specificity and was almost equal to a tablet app in a previous study, which diagnosed CTS with 93% sensitivity and 73% specificity [19]. The result was also as good as physical examinations; the Tinel sign showed 23% to 60% sensitivity and 64% to 87% specificity, and the Phalen test showed 51% to 91% sensitivity and 33% to 86% specificity in previous studies [11,12]. As we could obtain the same diagnostic ability as physical examinations without a direct medical examination, the app would be useful for screening for CTS in telemedicine in the circumstances of COVID-19.

In the modified screening models, the model using data from directions 8 to 11 had the highest AUC of 0.99 and could diagnose CTS with 92% sensitivity and 100% specificity; this was better than the screening model that used data in all directions. This result suggests that thumb movement from directions 8 to 11 is different between the CTS and non-CTS groups, contributing to the diagnosis of CTS. Reaching directions 8 to 11 requires a movement similar to thumb opposition, as in screen B of Figure 3, a movement that is impaired in people with CTS [3]. This difficulty with thumb opposition was apparent when using our system.

We used a similar app as in the previous study [19] but with 2 novel aspects. First, our system used a smartphone instead of a tablet. The usage rate of smartphones in Japan is approximately 80% in people aged 40 years or older, higher than that of tablets (40%) [21]. Since we intend to use this app as a screening tool for CTS, it should be accessible to many people. Therefore, it is important to use common equipment. Smartphones have been used in many medical studies because of their utility and universality [16]. Second, our system used anomaly detection algorithms (instead of a binary classification), which have been studied extensively in the detection of system failures in infrastructure and factories, malware detection, and computer vision [22]. Anomaly detection algorithms are also used in medicine, such as medical images [25,26], electrocardiograms [27], and remote medicine [28,29]. Although classification techniques are the most common approaches to anomaly detection, data sets often lack sufficient labeled anomalies. In such cases, unsupervised anomaly detection using statistical and machine learning is more promising. The binary classification used in the previous study requires 2 data sets, one from healthy people and one from patients. In contrast, anomaly detection algorithms require only data sets of healthy people. In general, large data sets are required to enhance machine learning. If our app is used widely, it will be easier to collect data sets from healthy people than patients with CTS. Thus, our system can be enhanced easily in the future.

### Limitations

This study has some limitations. First, the varied sizes of the participants’ hands were not considered. Healthy people with small hands who struggled to reach each direction may have been misdiagnosed with CTS. Second, because smartphone sizes vary, the level of difficulty depends on the model. Therefore, it is desirable to adjust the size of the circle in the game before playing according to the size of each player's fingers and the smartphone. Third, we used an inexpensive finger guide on the back of the smartphone to fix the hand. If special equipment is required, few people will be able to use our system. It would be better to use readily available equipment, such as fall prevention devices for smartphones, instead. Fourth, while we obtained good results in this study, there is still room for further improvement in machine learning. In order to take advantage of anomaly detection, it is desirable to collect more samples. Finally, our system diagnosed only the presence of CTS. In future work, we will improve our system by collecting more data sets to enable estimation of the severity of CTS.

### Conclusions

We developed an app for screening patients with CTS that revealed the difficulty of thumb opposition for patients with CTS and could screen for CTS with high sensitivity and specificity. The app can be used by many people because it is smartphone based, and the machine learning is easy to enhance using anomaly detection. In future work, we will enhance our system by collecting more data sets to enable estimation of the severity of CTS.

## Abbreviations

AE: autoencoder
AUC: area under the curve
CTS: carpal tunnel syndrome
NCS: nerve conduction study
ROC: receiver operating characteristic
